# Effectiveness of traditional mind-body exercises on fall risk test scores and balance in older adults: A systematic review and meta-analysis

**DOI:** 10.1097/MD.0000000000051150

**Published:** 2026-10-16

**Authors:** Li Wan, Yanxin Guo, Cen Liang

**Affiliations:** a The Sanya Rehabilitation and Recuperation Center of the PLA Joint Logistics Support Force, Sanya, Hainan, China.

**Keywords:** balance ability, fall risk test scores, meta-analysis, older adults, traditional mind-body exercises

## Abstract

**Background:**

This systematic review and meta-analysis aimed to evaluate the effects of traditional mind-body exercises on fall risk test scores and balance in older adults.

**Methods:**

The systematic search covered multiple databases, including PubMed, Cochrane Library, Embase, Web of Science, China National Knowledge Infrastructure (CNKI), Wanfang, and Weipu (VIP), with the search period spanning from the inception of each database to December 10, 2025. We included randomized controlled trials evaluating the effects of traditional mind-body exercises on fall risk test scores and balance in older adults. Study quality was assessed using the Cochrane Risk of Bias Tool. Meta-analysis was performed using Stata 15 software and a random-effects model.

**Results:**

A total of 31 randomized controlled trials involving 3622 participants were included. Pooled analyses showed lower fall risk test scores (standardized mean difference [SMD] = −0.56, 95% confidence interval [CI]: −0.84 to −0.28) and higher Berg Balance Scale scores (WMD = 2.60, 95% CI: 1.47–3.72) in the intervention groups than in the control groups. Heterogeneity was very high for Berg Balance Scale scores (*I*^2^ = 95.6%). Subgroup results for fall risk test scores were Baduanjin (BDJ) [SMD = −0.82, 95% CI (−1.25, −0.38)] and Tai Chi (TC) [SMD = −0.41, 95% CI (−0.70, −0.11)]. For Berg Balance Scale scores, results were BDJ [WMD = 2.91, 95% CI (1.02, 4.8)], TC [WMD = 2.37, 95% CI (0.83, 3.9)], and Wuqinxi (WQX) [weighted mean difference (WMD) = 4.80, 95% CI (1.40, 8.20)]. Examples of protocols used in the included trials included Tai Chi twice weekly for 55 minutes per session over 8 weeks, Baduanjin 3 times weekly for 40 minutes per session over 12 weeks, and Wuqinxi 4 times weekly for 60 minutes per session over 12 weeks.

**Conclusion:**

Low-certainty evidence suggests that Tai Chi and Baduanjin may reduce fall risk test scores and improve balance in older adults, while Wuqinxi may improve balance. These findings should be interpreted cautiously and do not establish a reduction in actual fall incidence.

## 1. Introduction

As the global population ages, health issues among the elderly have become a critical public health concern.^[1]^ According to United Nations data, by 2050, individuals aged 60 and above will constitute over 20% of the world’s population.^[2]^ This demographic shift presents multifaceted health challenges, with falls and their consequences emerging as one of the most prevalent and harmful health issues affecting older adults. Research indicates that approximately 28% to 35% of older adults worldwide experience a fall each year.^[3]^ Falls are not only a leading cause of fractures and death among the elderly but are also closely associated with multiple long-term health issues, including hip fractures, head injuries, prolonged bed rest, functional decline, and mental health problems.^[4,5]^

Falls often inflict dual physical and psychological trauma on the elderly. Following a hip fracture, approximately half of participants face extended recovery periods, with 15% to 20% succumbing to complications or prolonged bed rest.^[6]^ Furthermore, falls can instill a fear of falling, leading to reduced mobility and creating a vicious cycle that diminishes quality of life and independence.^[7]^ Research indicates that falls among the elderly result from multiple intertwined factors. Beyond physiological factors (such as diminished muscle strength, joint function deterioration, and reduced balance), environmental factors (obstacles in living spaces, slippery surfaces, inadequate lighting) and psychological factors (fear of falling) also play significant roles.^[8,9]^ Balance, which refers to an individual’s ability to maintain a stable posture while stationary or in motion,^[10]^ plays a crucial role in fall prevention but generally declines with advancing age. Reduced physical strength and coordination make older adults more susceptible to falls.^[11]^ Fall incidents among the elderly often result in severe injuries and long-term functional impairment. Therefore, improving balance is considered key to fall prevention.

In recent years, traditional mind-body exercises have received increasing attention as forms of physical activity for older adults. These practices emphasize coordinated movement, breathing, and relaxation.^[12]^ Previous research has examined their potential benefits for balance and outcomes related to falls.^[13-15]^ However, uncertainty remains regarding their effects on fall risk test scores and balance across different exercise modalities. Therefore, this systematic review and meta-analysis aimed to evaluate the effects of traditional mind-body exercises on fall risk test scores and Berg Balance Scale scores in older adults and to explore the findings across exercise types. Actual fall incidence was not evaluated in this meta-analysis.

## 2. Materials and methods

This systematic review and meta-analysis were reported in accordance with PRISMA (Preferred Reporting Items for Systematic Reviews and Meta-Analyses) guidelines.^[16]^ This study was preregistered with the International prospective register of systematic reviews (PROSPERO) under the registration number: CRD420261288757.

### 2.1. Ethical approval and informed consent

Ethical approval and informed consent were not required because this systematic review and meta-analysis used aggregate data from previously published studies and did not involve participant recruitment or access to identifiable individual-level data.

### 2.2. Inclusion criteria

*Study design*: All published randomized controlled trials (RCTs) examining the effects of traditional mind-body exercises on fall risk test scores and/or balance in older adults.

*Participants*: Healthy older adults aged ≥60 years, or older adults at high risk of falls (those with balance issues, gait disorders, or a history of falls).

*Interventions*: Traditional mind-body exercise interventions, including but not limited to TC (Tai Chi), WQX (Wuqinxi), BDJ (Baduanjin), PLT (Pilates).

*Comparison groups*: Any nonintervention or control group, including usual care, educational interventions, or other types of exercise interventions.

*Primary outcomes*: Fall risk test scores and Berg Balance Scale scores.

### 2.3. Exclusion criteria

*Study design*: Case reports, case-control studies, cross-sectional studies, review articles, conference abstracts, and non-peer-reviewed literature.

*Participants*: Elderly individuals under 60 years of age or with severe health conditions (severe cardiovascular disease, cognitive impairment) rendering them unsuitable for mind-body exercise.

*Interventions*: Interventions not classified as traditional mind-body exercises, such as strength training, endurance training, or other modern fitness methods.

*Comparison groups*: Studies lacking an effective control group or failing to clearly report the intervention content of the control group.

*Outcome measures*: Studies lacking valid quantitative data on fall risk test scores and/or balance, or employing ineffective or inappropriate outcome measurement tools.

*Data quality*: Studies failing to meet minimum quality standards, providing insufficient data, or employing inadequate analytical methods, thereby precluding valid meta-analysis.

### 2.4. Literature search

We searched PubMed, Cochrane Library, Embase, Web of Science, China National Knowledge Infrastructure (CNKI), Weipu (VIP), and Wanfang. The specific search strategy encompassed interventions (Mind-Body Therapies, Tai Chi, Qigong, Baduanjin, Liuzijue, Yijinjing), study populations (“older adults,” “elderly”), and combined keywords using Boolean operators (AND, OR, NOT). Standardized retrieval was achieved through Medical Subject Headings (MeSH) terms. The search period spanned from the inception of each database to December 10, 2025. The detailed search strategy is presented in Table S1, Supplemental Digital Content 1.

### 2.5. Data extraction

In this systematic review and meta-analysis, data extraction was conducted following the recommended process outlined in the PRISMA 2020 guidelines. Two independent researchers extracted data from the included studies based on predefined criteria. During data extraction, for each study, basic information was first recorded, including authors, publication year, sample size, and participants’ baseline characteristics (age, gender, health status). Subsequently, information regarding the intervention measures was extracted, including the type of traditional mind-body exercise, intervention duration, session frequency, and session duration. Additionally, primary outcome measures related to fall risk test scores and balance in older adults were extracted.

For outcomes concerning fall risk and physical balance ability, we specifically extracted the following data: fall risk scores and the Berg Balance Scale scores. When studies reported data from multiple follow-up time points, we selected the longest-term follow-up data for analysis. Where data were incomplete or unclear, we obtained missing information by contacting original authors or estimated values using appropriate statistical methods (imputation techniques). Discrepancies between 2 extractors were resolved through discussion, with third-party researchers arbitrating when necessary. All extracted data were recorded in Excel spreadsheets for subsequent statistical analysis.

### 2.6. Risk of bias

In this study, we used the Risk of bias ROB 2 tool^[17]^ to assess the quality of the included randomized controlled trials (RCTs). This tool evaluates the risk of bias across 5 key domains: randomization process, intervention implementation, missing data, measurement bias, and reporting bias. Quality assessments were conducted by 2 independent researchers, with any disagreements arbitrated by a third researcher. The risk of bias for each study was categorized as “low risk,” “high risk,” or “Some concerns.” All assessment results were discussed in the appendix, and the potential impact of risk of bias on study outcomes was discussed.

### 2.7. GRADE assessment

To systematically assess the quality of the evidence included in the study, this research used the GRADE (Grade of Recommendations Assessment, Development, and Evaluation)^[18]^ system to rate the final evidence. The GRADE system is a tool widely used in clinical research that comprehensively evaluates the quality of evidence by assessing factors such as study design, risk of bias, consistency, directness, and precision. According to the GRADE system, evidence quality is divided into 4 levels: high, moderate, low, and very low.

### 2.8. Data analysis

Statistical analyses were performed using Stata version 15.0 (StataCorp, College Station). Fall risk test scores were pooled using standardized mean differences (SMDs) with 95% confidence intervals (CIs), as the included studies assessed the same outcome using different measurement scales. Berg Balance Scale scores were pooled using weighted mean differences (WMDs) with 95% CIs because this outcome was measured using the same scale across studies. All meta-analyses, including subgroup analyses, were performed using random-effects models to account for between-study variability.

Statistical heterogeneity was assessed using Cochran *Q* test and quantified using the *I*^2^ statistic. *I*^2^ values of approximately 25%, 50%, and 75% were interpreted as indicating low, moderate, and high heterogeneity, respectively. Random-effects models were used irrespective of the observed degree of heterogeneity. Exploratory subgroup analyses were conducted according to the type of mind-body exercise. Leave-one-out sensitivity analyses were performed by sequentially excluding each study and recalculating the pooled effect estimate to assess the influence of individual studies and the robustness of the findings.

Funnel plots were visually inspected, and Egger regression test was used to explore small-study effects. These assessments were interpreted cautiously because funnel plot asymmetry can arise from factors other than publication bias. In particular, the assessment of fall risk test scores was considered exploratory because only 7 studies were available, limiting the power of the asymmetry test. The absence of statistically significant asymmetry was not considered evidence that publication bias was absent.

## 3. Results

As shown in Figure 1, PubMed (n = 1043), Embase (n = 1265), Cochrane Library (n = 885), Web of Science (n = 1628), CNKI (n = 20), Wanfang (n = 36), and VIP (n = 25) yielded a total of 4902 articles. After removing 1224 duplicate records, 3638 articles were excluded based on title and abstract screening, and 9 articles were excluded after full-text review. Ultimately, 31 articles^[19-49]^ were included for analysis.

**Figure 1.**
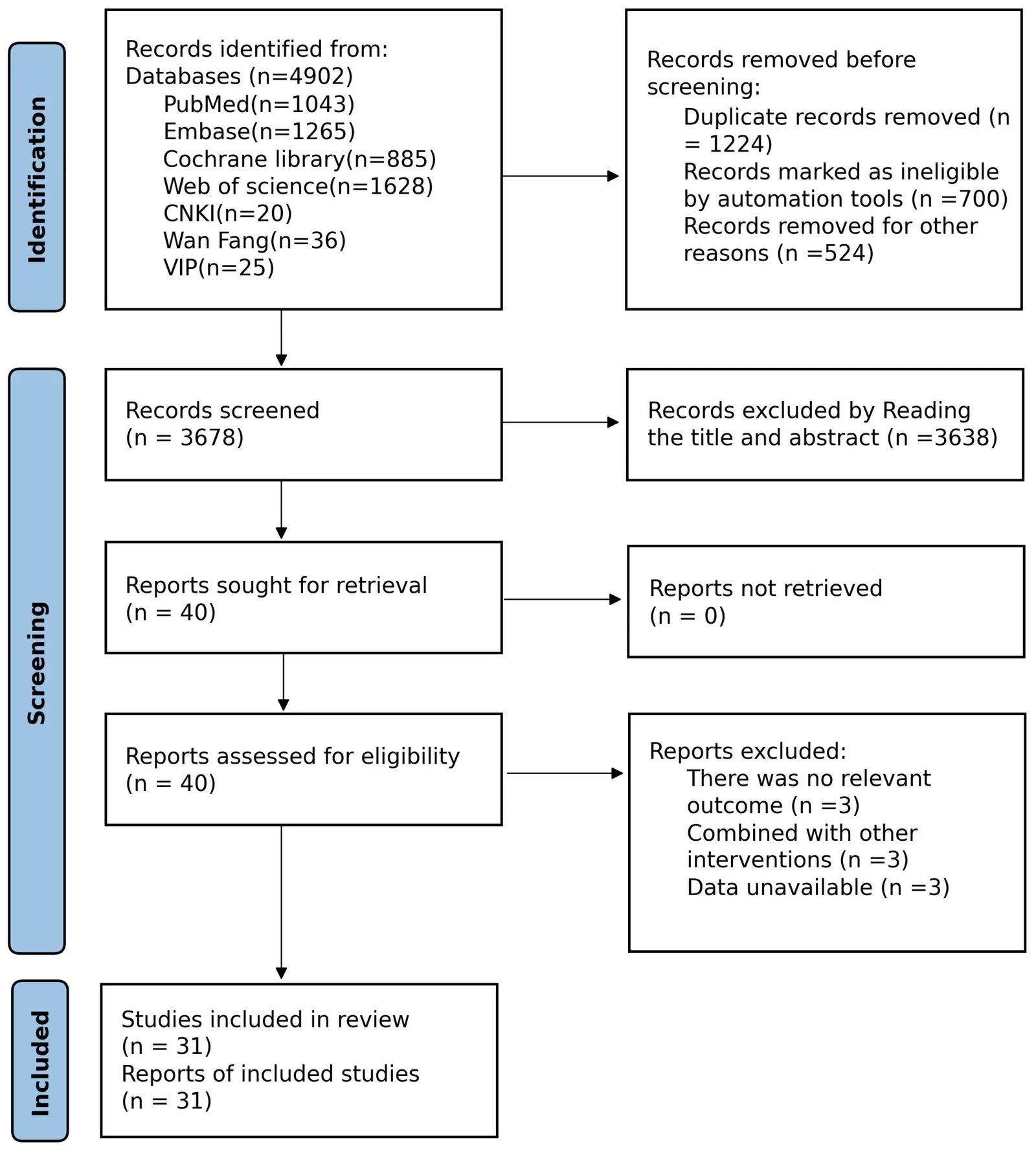
Literature search flow chart.

### 3.1. Basic characteristics of the included literature

This study included a total of 31 randomized controlled trials involving 3622 participants. Interventions comprised BDJ, TC, WQX, and PLT, with participants aged 60 to 80 years. Detailed baseline characteristics are presented in Table 1.

**Table 1:** Table of basic characteristics of the literature.

Study	Year	Country	Sample size	Gender (M/F)	Mean age (yr)	Comorbidity	Intervention	Outcomes
ZC Sun	2020	China	BDJ: 30 UT: 30	15/45	BDJ: 73.96 UT: 72.53	Osteoporosis	BDJ: 50 min/session; 3 sessions/wk; 12 mo	F1
ZY Ma	2022	China	TC: 50 UT: 45	NR	TC: 68.15 UT: 66.69	NR	TC: 30 min/session; 4 sessions/wk; 6 mo	F1; F3
WY Du	2018	China	BDJ: 94 UT: 63	92/108	BDJ: 65.36 UT: 67.21	NR	BDJ: 60 min/session; 5 sessions/wk; 6 mo	F3
XH Guan	2017	China	TC: 40 UT: 40	NR	NR	Parkinson	TC: 60 min/session; 5 sessions/wk; 6 mo	F3
BB Tong	2024	China	BDJ: 52 UT: 52	43/61	BDJ: 79.48 UT: 78.44	NR	BDJ: 20 min/session; 2 sessions/d; 28 d	F3
XY Shi	2017	China	BDJ: 52 UT: 52	53/51	BDJ: 64.7 UT: 65.5	NR	BDJ: 30–40 min/session; 3 sessions/day; 12 weeks	F3
C Wang	2019	China	BDJ: 42 UT: 42	46/38	BDJ: 66.4 UT: 66.6	psychiatric	BDJ: 30 min/session; 2 sessions/d; 12 wk	F3
D Hong	2018	China	TC: 37 UT: 35	37/35	TC: 59.23 UT: 69.97	NR	TC: 30 min/session; 1 session/wk; 6 mo	F1
ZF Li	2019	China	BDJ: 44 UT: 44	28/60	BDJ: 65.1 UT: 65.5	Senile osteoporosis	BDJ: 30–40 min/session; 24 wk	F3
CZ Peng	2023	China	TC: 45 UT: 43	88/0	TC: 63.6 UT: 63.1	NR	TC: 50 min/session; 1 session/d, 6 sessions/wk; 24 wk	F1
SP Zhou	2020	China	BDJ: 20 UT: 20	17/23	BDJ: 72.67 UT: 73.25	Sarcopenia	BDJ: 40 min/session; 1 session/d; 8 wk	F1; F3
J Zhou	2020	China	BDJ: 30 UT: 31	25/36	BDJ: 59.70 UT: 61.13	NR	BDJ: 30 min/session; 1 session/d, 5 sessions/wk; 3 mo	F3
X Wu	2017	China	BDJ: 60 UT: 60	36/84	BDJ: 70.63 UT: 70.55	NR	BDJ: 2 sessions/d; 30 d	F3
YN Zhao	2016	China	TC: 20 CON: 21	13/28	TC: 68.8 CON: 69.9	NR	TC: 90 min/session; 3 sessions/wk; 16 wk	F1
L Day	2015	Australia	TC: 204 UT: 205	152/351	TC: 77.6 UT: 77.8	NR	TC: 60 min/session; 2 sessions/wk; 48 wk	F1; F3
Birimoglu	2017	Turkey	TC: 20 UT: 24	23/21	NR	NR	TC: 35–40 min/session; 2 sessions/wk; 12 wk	F3
QY He	2025	China	TC: 17 UT: 19	13/23	TC: 57.4 UT: 58.6	Senile knee osteoarthritis	TC: 35–40 min/session; 4 sessions/wk; 8 wk	F3
L Hosseini	2018	Iran	TC: 30 UT: 30	33/27	NR	NR	TC: 55 min/session; 2 sessions/wk; 8 wk	F3
FZ Li	2012	United States	TC: 65 UT: 65	84/46	TC: 68 UT: 5869	Parkinson	TC: 60 min/session; 2 sessions/wk; 24 wk	F1
CM Xiao	2021	China	WQX: 34 UT: 34	45/23	WQX: 70.7 UT: 70.2	knee osteoarthritis	WQX: 60 min/session; 4 sessions/wk; 12 wk	F3
M Mao	2024	United States	TC: 40 Stretching: 31	17/42	TC: 78.3 Stretching: 79.9	NR	TC: 60 min/session; 4 sessions/wk; 16 wk	F3
J Pan	2025	China	TC: 12 UT: 12	7/17	TC: 65.17 UT: 62.89	Knee osteoarthritis	TC: 60 min/session; 3 sessions/wk	F3
S Xie	2025	China	BDJ: 22 UT: 20	21/21	BDJ: 64.6 UT: 63.7	NR	BDJ: 40 min/session; 3 sessions/wk; 12 wk	F1
J. Song	2022	China	TC: 20 UT: 20	0/40	TC: 64.15 UT: 64.15	Knee osteoarthritis	TC: 60 min/session; 3 sessions/wk; 12 wk	F3
GL Xie	2018	China	TC: 120 UT: 124	169/75	TC: 60.9 UT: 60.1	NR	TC: 60 min/session; 5 sessions/wk	F3
M Roller	2018	United States	PLT: 27 UT: 28	17/38	PLT: 78.52 UT: 76.68	NR	PLT: 45 min/session; 1 session/wk; 10 wk	F3
IH Logghe	2009	Netherlands	TC: 138 UT: 131	78/191	TC: 77.5 UT: 76.8	NR	TC: 60 min/session; 2 sessions/wk; 13 wk	F3
CM Xiao	2016	China	BDJ: 48 UT: 48	67/29	BDJ: 68.17 UT: 66.52	NR	BDJ: 45 min/session; 4 sessions/wk; 6 mo	F3
Yildirim	2025	Turkey	TC: 30 UT: 30	25/35	TC: 72.07 UT: 70.97	NR	TC: 60 min/session; 2 sessions/wk; 15 wk	F3
LL Duan	2024	China	BDJ: 523 UT: 505	297/731	BDJ: 68.32 UT: 70.44	NR	BDJ: 45 min/session; 4 sessions/wk; 6 mo	F3

BDJ = Baduanjin, CON = control group, F1 = Fall Risk Test, F3 = Berg Balance Scale scores, M/F = male/female, min = minutes, NR = not reported, PLT = Pilates, TC = Tai Chi, UT = usual treatment, WQX = Wuqinxi.

### 3.2. Risk of bias results

This study employed the Risk of Bias 2.0 tool for quality assessment. Results (Figs. S1 and S2, Supplemental Digital Content 2) indicate that among the 31 included studies, 22 reported their randomization methods, whereas the remaining 9 provided insufficient details. Using the GRADE approach (Table S2, Supplemental Digital Content 3), the certainty of evidence was low-quality for fall risk test scores and very low for Berg Balance Scale scores.

### 3.3. Meta-analysis results

#### 3.3.1. Fall risk test scores

Seven studies reported on fall risk test scores, with significant heterogeneity (*I*^2^ = 56.2%, *P* = .033). Analysis using a random-effects model (Fig. 2) indicated that traditional mind-body exercises reduced fall risk test scores in older adults [SMD = -0.56, 95% CI (−0.84, −0.28)]. Sensitivity analysis (Fig. S3, Supplemental Digital Content 4) indicated robust results unaffected by any single study. Subgroup analysis by intervention type (Fig. 3) showed that BDJ [SMD = −0.82, 95% CI (−1.25, −0.38)] and TC [SMD = −0.41, 95% CI (−0.70, −0.11)] were associated with lower fall risk test scores.

**Figure 2.**
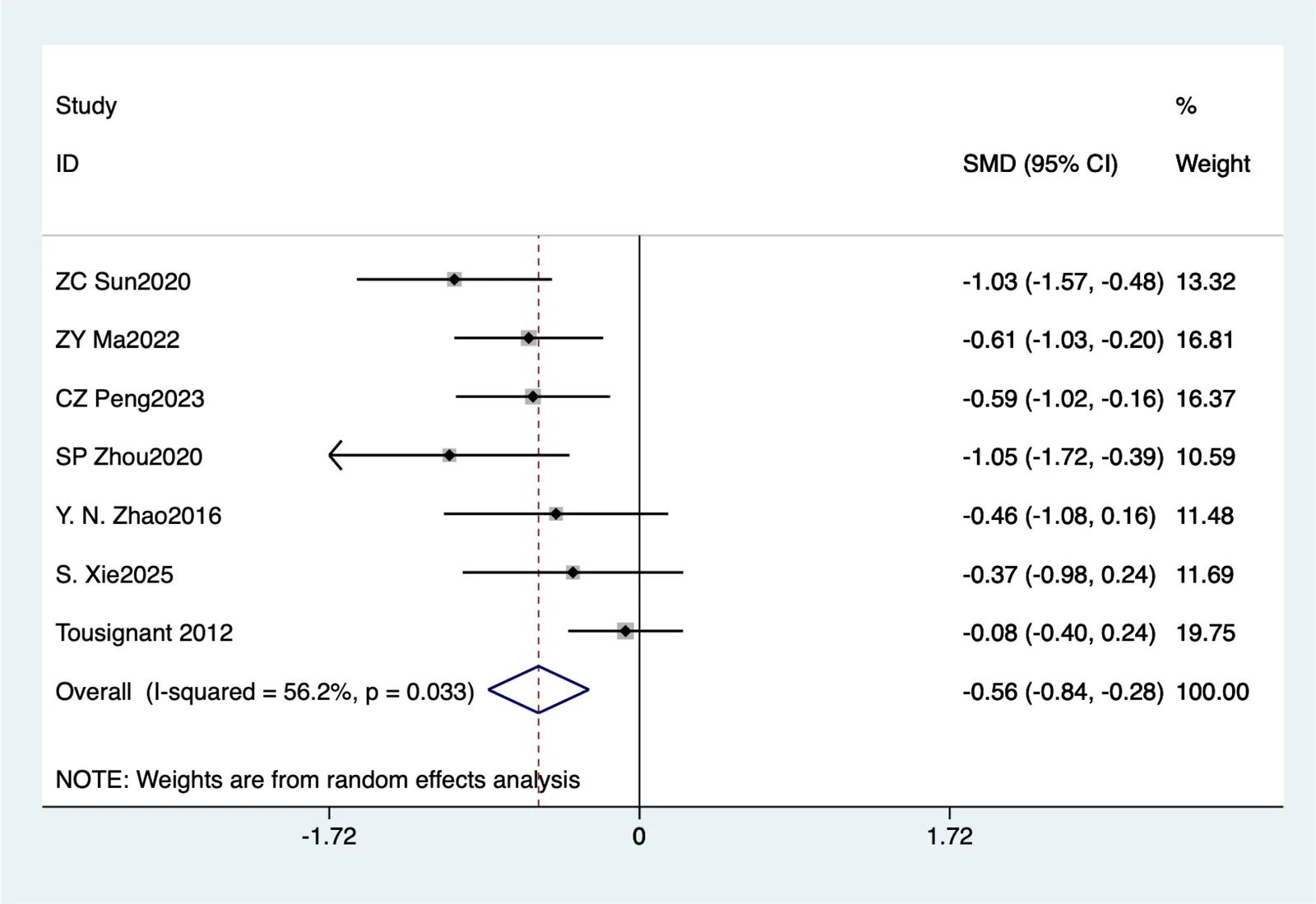
Forest plot of the meta-analysis of fall risk test scores. CI = confidence interval, SMD = standardized mean difference.

**Figure 3.**
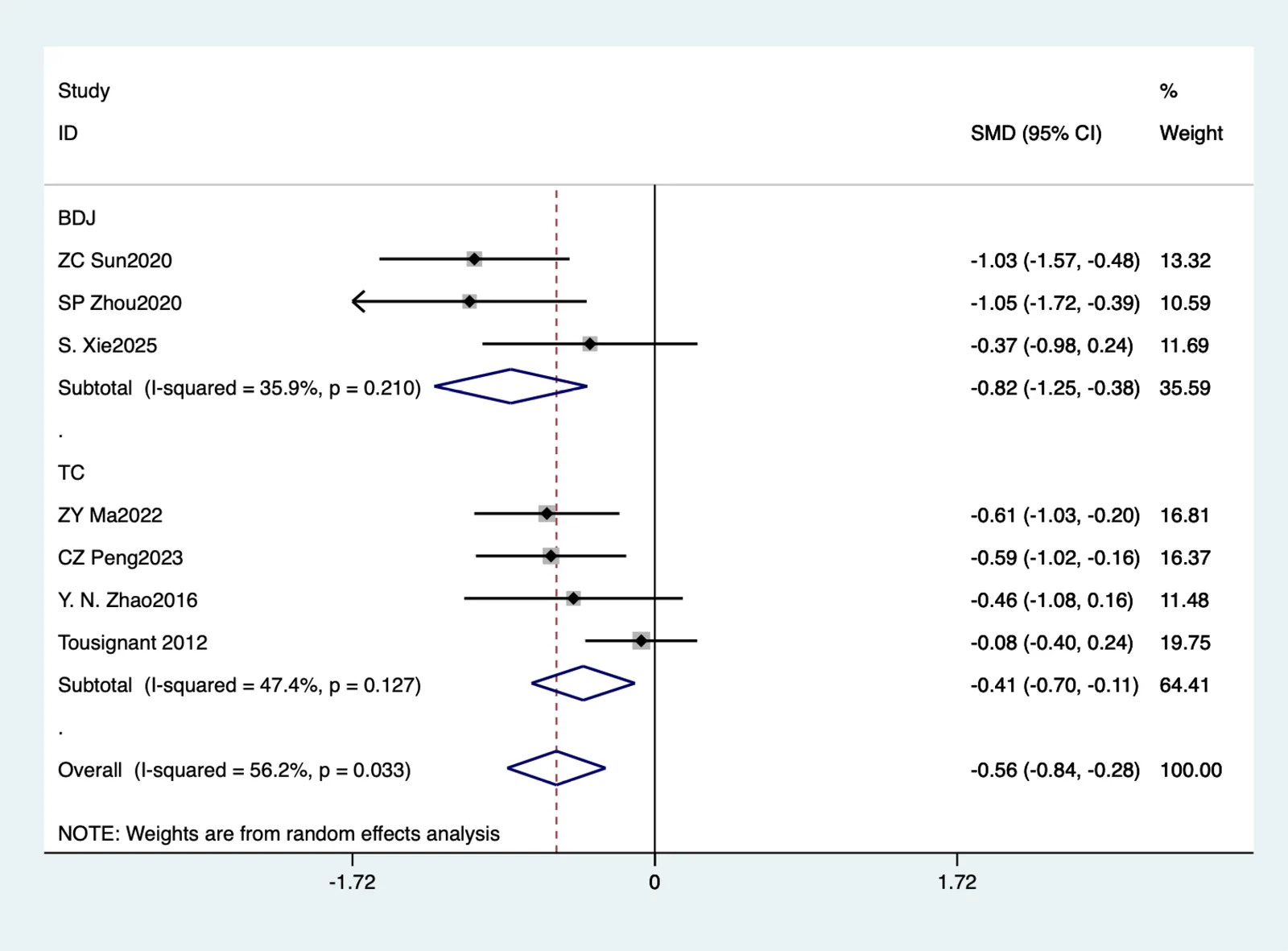
Forest plot of the subgroup meta-analysis of fall risk test scores. BDJ = Baduanjin, CI = confidence interval, SMD = standardized mean difference, TC = Tai Chi.

#### 3.3.2. Berg Balance Scale scores

Twenty-four studies reported Berg Balance Scale scores. The pooled analysis (Fig. 4) using a random-effects model showed higher Berg Balance Scale scores in the intervention groups than in the control groups (WMD = 2.60, 95% CI (1.47, 3.72)), with very high heterogeneity (*I*^2^ = 95.6%, *P* = .001). Leave-one-out sensitivity analyses (Fig. S4, Supplemental Digital Content 5) indicated that no single study substantially influenced the pooled estimate. However, this finding does not resolve concerns about heterogeneity or increase the certainty of the evidence. Exploratory subgroup analyses by intervention type (Fig. 5) showed that BDJ [WMD = 2.91, 95% CI (1.02, 4.8)], TC [WMD = 2.37, 95% CI (0.83, 3.9)], and WQX [WMD = 4.8, 95% CI (1.4, 8.2)] were associated with higher Berg Balance Scale scores.

**Figure 4.**
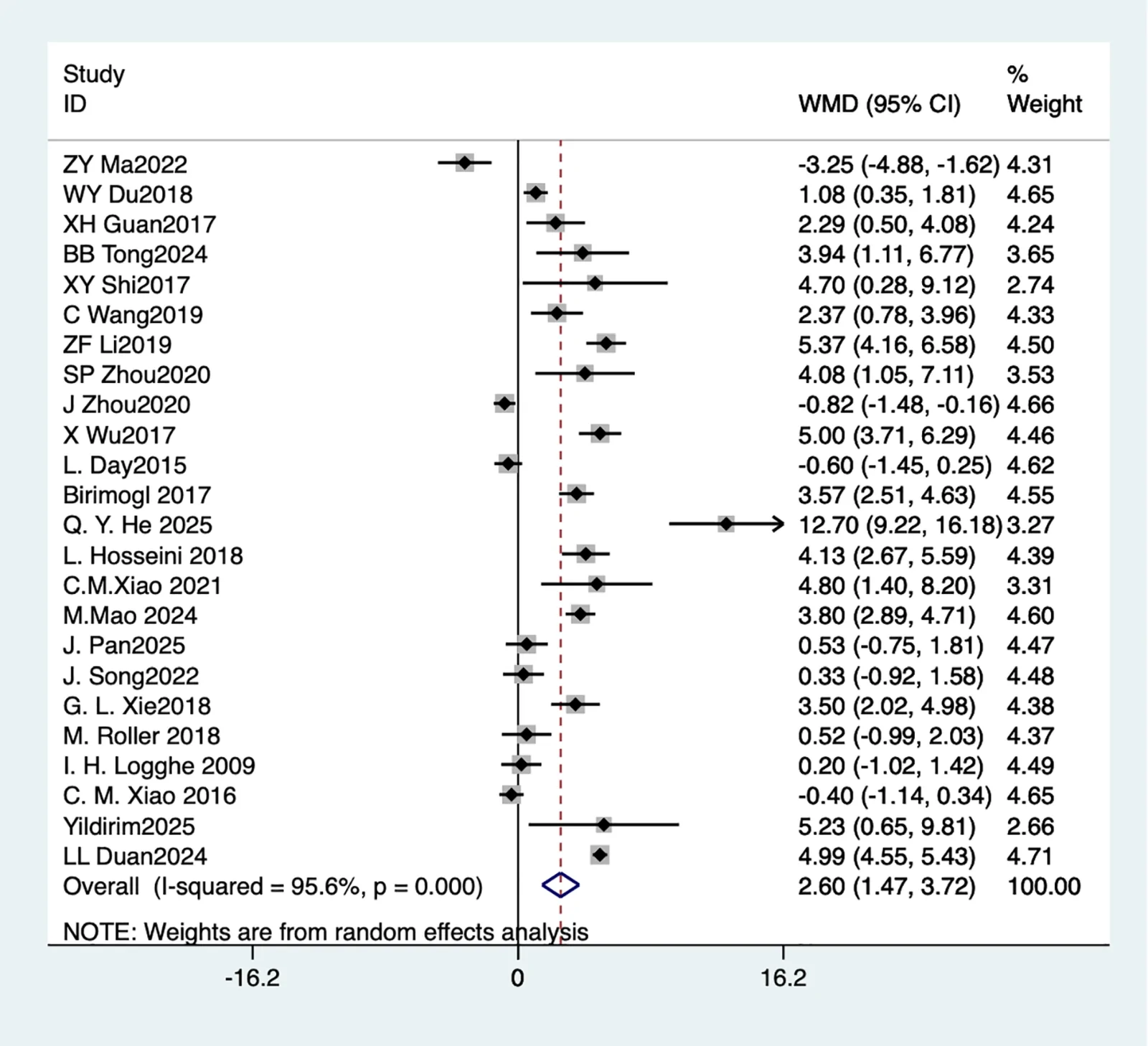
Forest plot of the meta-analysis of Berg Balance Scale scores. CI = confidence interval, WMD = weighted mean difference.

**Figure 5.**
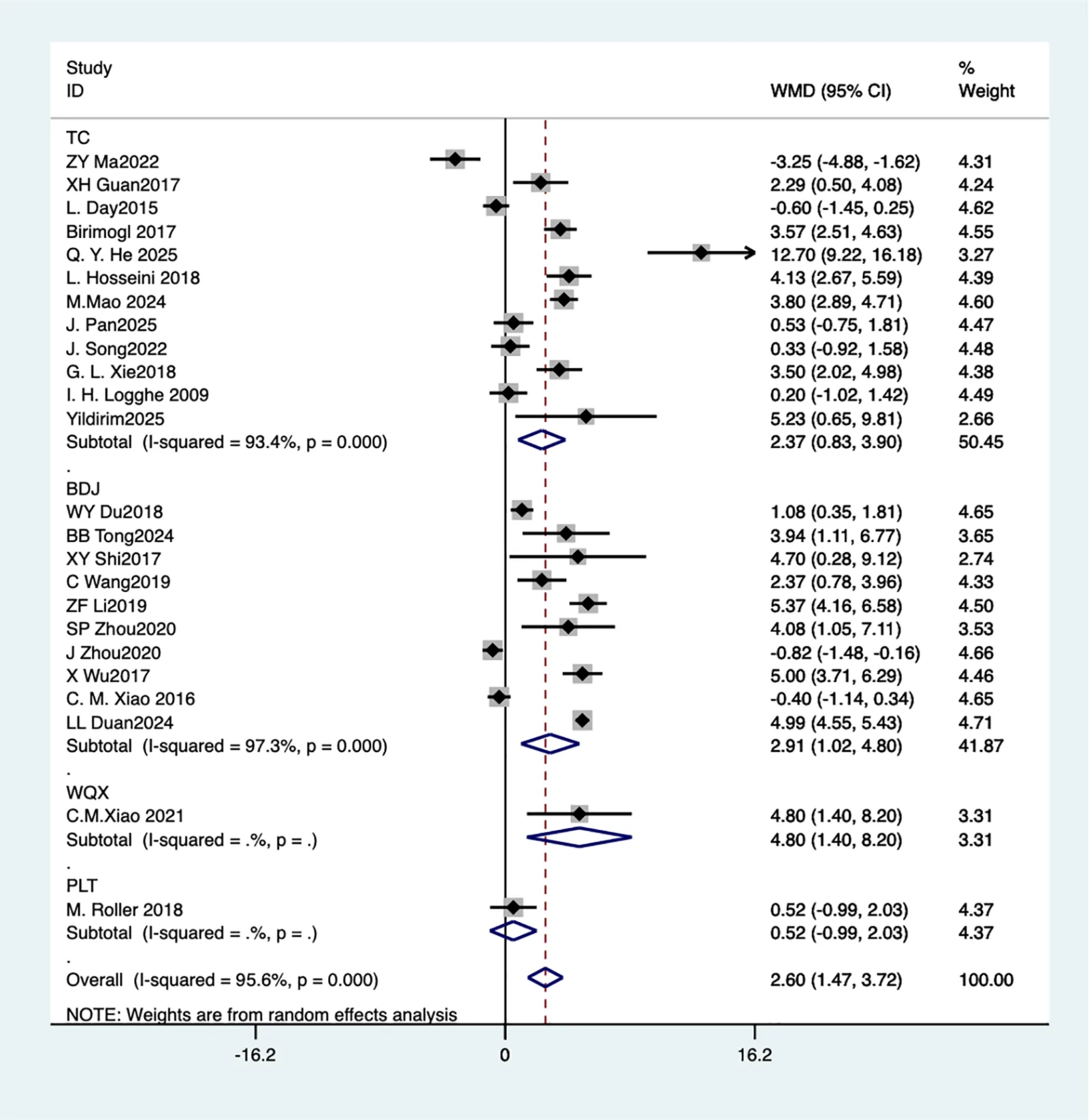
Forest plot of the subgroup meta-analysis of Berg Balance Scale scores. BDJ = Baduanjin, CI = confidence interval, PLT = pilates, TC = Tai Chi, WMD = weighted mean difference, WQX = Wuqinxi.

### 3.4. Publication bias

This study employed funnel plots and Egger test to assess publication bias. Results (Figs. S5 and S6, Supplemental Digital Content 6) indicate that funnel plots for fall risk test scores (Egger *P* = .092) and Berg Balance Scale scores (Egger *P* = .914) were symmetrical. Egger tests did not detect statistically significant asymmetry; however, publication bias cannot be excluded, particularly for fall risk test scores, for which only 7 studies were available.

## 4. Discussion

The findings of this systematic review and meta-analysis suggest that traditional mind-body exercises may improve fall risk test scores and Berg Balance Scale scores in older adults. Subgroup analyses suggested lower fall risk test scores for BDJ and TC and higher Berg Balance Scale scores for BDJ, TC, and WQX. These findings should be interpreted cautiously because the certainty of evidence was low for both outcomes, and substantial heterogeneity was observed, particularly for the Berg Balance Scale scores (*I*^2^ = 95.6%). Improvements in these test scores do not establish a reduction in actual fall incidence.

Seven studies were included in the meta-analysis of fall risk test scores. The pooled analysis showed lower fall risk test scores in the intervention groups than in the control groups, with heterogeneity across studies (*I*^2^ = 56.2%). Sensitivity analysis indicated that no single study substantially influenced the pooled estimate. Subgroup analyses also showed lower fall risk test scores for BDJ and TC. These findings suggest potential benefits of mind-body exercises for fall risk test scores. However, the low certainty of evidence limits confidence in the estimated effects.^[50,51]^ Because this meta-analysis evaluated fall risk test scores rather than actual fall events,^[52]^ the findings do not demonstrate reduced fall incidence or establish the effectiveness of these interventions for preventing falls.^[53]^

The pooled analysis showed higher Berg Balance Scale scores in the intervention groups than in the control groups. However, substantial heterogeneity was observed (*I*^2^ = 95.6%, *P* = .001), indicating considerable variability across studies and limiting the generalizability of the pooled estimate. Sensitivity analysis indicated that no single study substantially influenced the pooled estimate, but this finding does not resolve concerns about heterogeneity or establish broad applicability.^[54]^ Subgroup analyses showed higher Berg Balance Scale scores for BDJ, TC, and WQX. These subgroup findings should be interpreted cautiously and do not establish the superiority of one exercise modality over another.^[55,56]^ Overall, the low certainty of evidence limits confidence in the estimated benefits for balance.

## 5. Clinical significance

With the growing elderly population, falls and related health issues have become a major global public health challenge. According to the World Health Organization, an estimated 684,000 people die from falls worldwide each year, making falls a significant factor affecting their quality of life and health status. According to our findings, it appears that mind-body exercises may reduce fall risk test scores and improve Berg Balance Scale scores in older adults. The clinical importance of the observed difference remains uncertain.

## 6. Strengths and limitations

A strength of this systematic review and meta-analysis is the synthesis of evidence from 24 studies reporting Berg Balance Scale scores and 7 studies reporting fall risk test scores. Random-effects models were used to account for between-study variability, while sensitivity analyses examined the influence of individual studies on the pooled estimates. Subgroup analyses provided exploratory findings across exercise types. These approaches support the interpretation of the available evidence but do not eliminate heterogeneity, increase the certainty of evidence, or establish comparative effectiveness between exercise modalities.

Several limitations should be considered. First, substantial heterogeneity was observed for the Berg Balance Scale scores (*I*^2^ = 95.6%), which may reflect differences in participant characteristics, intervention protocols, and study methods. Although random-effects models account for between-study variability, they do not remove heterogeneity, and the potential applicability of the pooled findings remains limited. Second, the certainty of evidence was rated as low for both fall risk test scores and very low for Berg Balance Scale scores according to GRADE, limiting confidence in the estimated effects. Third, the included studies generally had small sample sizes, and variations in exercise frequency and intensity may have contributed to differences in outcomes. Fourth, this meta-analysis assessed fall risk test scores and balance scores rather than actual fall incidence, so the findings cannot establish a preventive effect on falls. Finally, insufficient long-term follow-up evidence limits conclusions about the persistence of any benefits.

## 7. Conclusion

This study suggests that TC and BDJ may reduce fall risk test scores and improve balance in older adults, while WQX may improve balance. Representative protocols included TC twice weekly for 55 minutes per session over 8 weeks, BDJ 3 times weekly for 40 minutes per session over 12 weeks, and WQX 4 times weekly for 60 minutes per session over 12 weeks. These protocols represent examples from the included studies, and the optimal exercise dose remains uncertain. The findings should be interpreted cautiously because of the very high heterogeneity in Berg Balance Scale scores (*I*^2^ = 95.6%) and the very low certainty of evidence. Improvements in these test scores do not establish a reduction in actual fall incidence. Future research should further explore optimal intervention strategies for different mind-body exercises and validate their effects in larger and more diverse populations. Additionally, how to implement these interventions in real-world settings, particularly in promoting mind-body exercises among older adults, will be a key direction for future studies.

## Author contributions

**Conceptualization:** Li Wan, Yanxin Guo, Cen Liang.

**Data curation:** Li Wan, Yanxin Guo, Cen Liang.

















## References

[1] Tamakloe R Khorasani M Kim I. Differences in injury severities between elderly and non-elderly taxi driver at-fault crashes: temporal instability and out-of-sample prediction. Accid Anal Prev. 2025;211:107865. 10.1016/j.aap.2024.107865 39647232

[2] Yu G Xie W Xiang J. Gut microbiota remodelling alleviates elderly sepsis by microbiota-derived acetic acid via FFAR2/NLRP3 pathway. Eur J Pharmacol. 2025;1007:178229. 10.1016/j.ejphar.2025.178229 41072839

[3] Zheng L Wu X Gu W. Development and validation of a hypoxemia prediction model in middle-aged and elderly outpatients undergoing painless gastroscopy. Sci Rep. 2025;15:17965. 10.1038/s41598-025-02540-8 PMC12102271 40410303

[4] Ellmers TJ Ventre JP Freiberger E. Does concern about falling predict future falls in older adults? A systematic review and meta-analysis. Age Ageing. 2025;54:1–10. 10.1093/ageing/afaf089 PMC11976718 40197783

[5] Rommers E De Pauw R Petrovic M Cambier D. Epidemiology of falls in community-dwelling older adults in Europe: a systematic review and meta-analysis. Age Ageing. 2025;54:afaf157. 10.1093/ageing/afaf157 40479612

[6] Asano K Kabasawa K Takachi R. Association of dietary calcium intake with risk of falls in community-dwelling middle-aged and older adults. J Nutr Health Aging. 2025;29:100465. 10.1016/j.jnha.2024.100465 PMC12180026 39742575

[7] Li W Zhao M Fu Y. Performance of risk assessment algorithms recommended by the World Falls Guidelines (WFG) to predict fall and fall-related injury among older Chinese community-dwellers. J Safety Res. 2025;95:338–44. 10.1016/j.jsr.2025.10.018 41338789

[8] Alkeridy WA Alharbi KK Sofi SM. Risk factors and locations of falls among older pilgrims during Hajj: implications for targeted prevention. J Infect Public Health. 2025;18:102874. 10.1016/j.jiph.2025.102874 40614683

[9] Yang X Li W He J. A cross-sectional study on the epidemiology and risk factors for falls among the elderly in Chongqing based on the China elderly fall surveillance initiative. Sci Rep. 2025;15:43894. 10.1038/s41598-025-27327-9 PMC12708847 41397988

[10] Jiang J Zhou Q Zhang C Cong K. Activities of daily living mediate the association between balance and falls in middle aged and older adults. Sci Rep. 2025;15:28694. 10.1038/s41598-025-14608-6 PMC12328578 40770416

[11] Ibharokhonre AO Oseni TIA Fuh FN Adewuyi BO Iyalomhe ES. Evaluation of risk factors for falls among elderly patients attending a teaching hospital in southern Nigeria: a cross-sectional study. Sci Rep. 2025;15:33790. 10.1038/s41598-025-97471-9 PMC12484555 41028799

[12] Lipardo DS Aseron AMC Kwan MM Tsang WW. Effect of exercise and cognitive training on falls and fall-related factors in older adults with mild cognitive impairment: a systematic review. Arch Phys Med Rehabil. 2017;98:2079–96. 10.1016/j.apmr.2017.04.021 28554873

[13] Zhu TR Xu HQ Wei JP. Effectiveness of exercise prescription variables to reduce fall risk among older adults: a meta-analysis. Eur Rev Aging Phys Act. 2025;22:7. 10.1186/s11556-025-00374-x PMC12070723 40360991

[14] Lyu J Li W Rong X. Efficacy of practising Tai Chi for older people with mild dementia: protocol for a randomised controlled study. BMJ Open. 2018;8:e019940. 10.1136/bmjopen-2017-019940 PMC5961586 29764877

[15] Choudhary A Pathak A Manickam P. Effect of yoga versus light exercise to improve well-being and promote healthy aging among older adults in Central India: a study protocol for a randomized controlled trial. Geriatrics (Basel). 2019;4:64. 10.3390/geriatrics4040064 PMC6960920 31744171

[16] Page MJ McKenzie JE Bossuyt PM. The PRISMA 2020 statement: an updated guideline for reporting systematic reviews. BMJ. 2021;372:n71. 10.1136/bmj.n71 PMC8005924 33782057

[17] Lu T Lu C Li H. The reporting quality and risk of bias of randomized controlled trials of acupuncture for migraine: methodological study based on STRICTA and RoB 2.0. Complement Ther Med. 2020;52:102433. 10.1016/j.ctim.2020.102433 32951707

[18] Guyatt GH Oxman AD Vist GE.; GRADE Working Group. GRADE: an emerging consensus on rating quality of evidence and strength of recommendations. BMJ. 2008;336:924–6. 10.1136/bmj.39489.470347.AD PMC2335261 18436948

[19] Birimoglu Okuyan C Bilgili N. Effect of Tai Chi Chuan on fear of falling, balance and physical self-perception in elderly: a randomised controlled trial. Turk Geriatri Derg. 2017;20:232–41.

[20] Day L Hill KD Stathakis VZ. Impact of Tai-Chi on falls among preclinically disabled older people. a randomized controlled trial. J Am Med Dir Assoc. 2015;16:420–6. 10.1016/j.jamda.2015.01.089 25769960

[21] He QY Wang F. Taichi is medicine: effects of Taichi exercise on knee fitness and psychological health in older adults with knee osteoarthritis (KOA): A randomized controlled trial. Medicine (Baltimore). 2025;104:e44612. 10.1097/MD.0000000000044612 PMC12459455 40988215

[22] Hosseini L Kargozar E Sharifi F Negarandeh R Memari AH Navab E. Tai Chi Chuan can improve balance and reduce fear of falling in community dwelling older adults: a randomized control trial. J Exerc Rehabil. 2018;14:1024–31. 10.12965/jer.1836488.244 PMC6323335 30656165

[23] Hwang HF Chen SJ Lee-Hsieh J Chien DK Chen CY Lin MR. Effects of home-based Tai Chi and lower extremity training and self-practice on falls and functional outcomes in older fallers from the emergency department-a randomized controlled trial. J Am Geriatr Soc. 2016;64:518–25. 10.1111/jgs.13952 26865039

[24] Li FZ Harmer P Fitzgerald K. Tai Chi and postural stability in patients with Parkinson’s disease. N Engl J Med. 2012;366:511–9. 10.1056/NEJMoa1107911 PMC3285459 22316445

[25] Li KQ Yu HL Kortas JA Lin XJ Lipowski M. The effect of 12 weeks of Baduanjin exercise on cognitive function, lower limb balance and quality of life of the elderly with mild cognitive impairment: a randomized controlled trial. Gazz Med Ital Arch Sci Med. 2022;181:811–23.

[26] Logghe IH Zeeuwe PE Verhagen AP. Lack of effect of Tai Chi Chuan in preventing falls in elderly people living at home: a randomized clinical trial. J Am Geriatr Soc. 2009;57:70–5. 10.1111/j.1532-5415.2008.02064.x 19054193

[27] Mao M Mercer VS Li FZ Gross MT Blackburn T Yu B. The effect of Tai Chi lower extremity exercise on the balance control of older adults in assistant living communities. BMC Complement Med Ther. 2024;24:1–7. 10.1186/s12906-024-04382-9 PMC10918873 38448853

[28] Pan J Xie ZH Ye ST. The effects of Tai Chi on clinical outcomes and gait biomechanics in knee osteoarthritis patients: a pilot randomized controlled trial. Sci Rep. 2025;15:10–19. 10.1038/s41598-025-03943-3 PMC12117020 40425665

[29] Roller M Kachingwe A Beling J Ickes DM Cabot A Shrier G. Pilates Reformer exercises for fall risk reduction in older adults: a randomized controlled trial. J Bodyw Mov Ther. 2018;22:983–98. 10.1016/j.jbmt.2017.09.004 30368346

[30] Song J Wei L Cheng K. The effect of modified Tai Chi exercises on the physical function and quality of life in elderly women with knee osteoarthritis. Front Aging Neurosci. 2022;14:860762. 10.3389/fnagi.2022.860762 PMC9204295 35721018

[31] Xiao CM Li JJ Kang Y Zhuang YC. Follow-up of a Wuqinxi exercise at home programme to reduce pain and improve function for knee osteoarthritis in older people: a randomised controlled trial. Age Ageing. 2021;50:570–5. 10.1093/ageing/afaa179 32931545

[32] Xiao CM Zhuang YC. Effect of health Baduanjin Qigong for mild to moderate Parkinson’s disease. Geriatr Gerontol Int. 2016;16:911–9. 10.1111/ggi.12571 26310941

[33] Xie S Meng C Sakipova Z Shaharudin S. The effects of Baduanjin intervention on balance, lower limb strength, gait biomechanics and risks of fall among elderly. Prev Med Rep. 2025;55:103129. 10.1016/j.pmedr.2025.103129 PMC12171621 40529597

[34] Yildirim A Hosbay Z Ucgun H. The effect of Baduanjin exercise training on balance and fall risk in elderly individuals: a randomized controlled trial. Medicine (Baltimore). 2025;104:e42360. 10.1097/MD.0000000000042360 PMC12055094 40324224

[35] Zhao YN Chung PK Tong TK. Effectiveness of a community-based exercise program on balance performance and fear of falling in older nonfallers at risk for falling: a randomized, controlled study. J Aging Phys Act. 2016;24:516–24. 10.1123/japa.2015-0224 26796916

[36] Wu X Xue W Fang J. Eight-section qigong exercise intervention on the risk of falls in community elderly: a study of 60 cases. Chin J Mod Telemed Tradit Chin Med. 2017;15:106–9.

[37] Zhou J Zhao Y Wei M. Research on the effects of eight-section qigong on the balance ability, fall risk and lower limb surface electromyography of the elderly. Zhongzhen Guoyi Guoyao. 2020;31:124–6.

[38] Zhou S Zou Y Sun X Xu Q. Research on the prevention of falls in elderly people with muscle atrophy by eight-section qigong. Sports Sci. 2020;41:27–8, 30.

[39] Sun Z Gu X Ouyang G. Research on the effect of virtual reality-based eight-section qigong on the fall risk and quality of life of elderly patients with osteoporosis in nursing homes. China Med J Pharm. 2020;17:90–3, 7.

[40] Peng C Liu H Chen J Hua B Han L. Effects of disturbing Taijiquan on muscle strength, gait characteristics and anti-fall risk index of elderly men. Chinese Geriatr J. 2023;43:839–44.

[41] Li Z Deng Q Zhang Y. Observation on the therapeutic effect of eight-section qigong on the balance ability and fall risk of elderly patients with osteoporosis. Western Chin Med. 2019;32:62–5.

[42] Duan L Zhao Y Er Y. Cluster randomized controlled trial study on the effect of eight-section qigong on the balance function of community elderly. Chin J Epidemiol. 2024;45:250–6.

[43] Du H Xu J Lin M Zhang J Ye H. Research on the effect of simplified Taijiquan exercise on reducing the fear of falling in community elderly. Chin J Nurs. 2018;53:1224–9.

[44] Wang C Han F Li L Liu C Peng M. Application of eight-section qigong therapeutic method in preventing falls in elderly patients in mental hospitals. Chin J Integr Tradit Western Med (in Chinese and English). 2019;5:68–71.

[45] Shi X. Research on the effect of eight-section qigong exercise therapy on preventing falls in the elderly. Mod Diagn Treat. 2017;28:1–5.

[46] Tong B Chen Q. Research on the effect of balance exercise routine combined with eight-section qigong in reducing the fall risk of elderly patients. Zhejiang J Tradit Chin Med. 2024;59:797–8.

[47] Guan X Wu H Liu G Yi G Zhai S. The effect of Taijiquan training on balance function and fear of falling in early Parkinson’s disease patients. Jiangxi Med J. 2017;52:1124–7.

[48] Du W Su S Zhao Y. Clinical observation on improving the balance function and gait of elderly people by eight-section qigong. Henan J Tradit Chin Med. 2018;40:987–90.

[49] Ma Z Ma H Yang X Liu J Ran L Ding X. The effect of core muscle strength combined with Taijiquan exercise on the walking ability and fall risk of elderly people. Ningxia Med J. 2022;44:237–40.

[50] Barrado-Martín Y Heward M Polman R Nyman SR. Adherence to the class-based component of a Tai Chi exercise intervention for people living with dementia and their informal carers. J Aging Phys Act. 2021;29:721–34. 10.1123/japa.2020-0119 PMC7611713 33395630

[51] Benedetti MG Furlini G Zati A Letizia Mauro G. The effectiveness of physical exercise on bone density in osteoporotic patients. Biomed Res Int. 2018;2018:4840531. 10.1155/2018/4840531 PMC6323511 30671455

[52] Brown LM Sawyer LM Lensing SY. An outpatient Tai Chi program: effects on veterans’ functional outcomes. Nurs Forum. 2021;56:448–52. 10.1111/nuf.12532 33241873

[53] Buttolph L Corn J Hanes D Bradley R Senders A. Community qigong for people with multiple sclerosis: a pragmatic feasibility study. J Altern Complement Med. 2021;27:506–14. 10.1089/acm.2020.0481 PMC9030260 33769837

[54] Cabrolier-Molina J Martín-Rodríguez A Clemente-Suárez VJ. The effects of exercise intervention in older adults with and without sarcopenia: a systematic review. Sports (Basel). 2025;13:152. 10.3390/sports13050152 PMC12115393 40423288

[55] Cai HJ Zhang KA Wang MZ Li XM Ran F Han YB. Effects of mind-body exercise on cognitive performance in middle-aged and older adults with mild cognitive impairment: a meta-analysis study. Medicine (Baltimore). 2023;102:e34905. 10.1097/MD.0000000000034905 PMC10470775 37653776

[56] Carcelen-Fraile MDC Aibar-Almazan A Martinez-Amat A. Qigong for muscle strength and static postural control in middle-aged and older postmenopausal women: a randomized controlled trial. Front Med (Lausanne). 2021;8:784320. 10.3389/fmed.2021.784320 PMC8692287 34957157

