# Supplementary material for: Effectiveness of traditional mind-body exercises on fall risk test scores and balance in older adults: A systematic review and meta-analysis
Source: Medicine (Baltimore). 2026 Oct 16;105(42):e51150. doi: 10.1097/MD.0000000000051150 (PMC13681109; doi:10.1097/MD.0000000000051150)
Supplement: Supplementary file 1 [file medi-105-e51150-s001.docx]

**Table S1 detailed search strategy**

((("Aged"[Mesh]) OR ((((Aged[Title/Abstract]) OR (Elderly[Title/Abstract])) OR (Older[Title/Abstract])) OR (Geriatrics[Title/Abstract]))) AND (("Mind-Body Therapies"[Mesh]) OR ((((((((((((((((((((((Mind-Body Therapies[Title/Abstract]) OR (Mind Body Therapies[Title/Abstract])) OR (Mind-Body Therapy[Title/Abstract])) OR (Therapies, Mind-Body[Title/Abstract])) OR (Therapy, Mind-Body[Title/Abstract])) OR (Mind-Body Medicine[Title/Abstract])) OR (Mind Body Medicine[Title/Abstract])) OR (Tai Ji[Title/Abstract])) OR (Tai-ji[Title/Abstract])) OR (Tai Chi[Title/Abstract])) OR (Chi, Tai[Title/Abstract])) OR (Tai Chi Chuan[Title/Abstract])) OR (Taiji[Title/Abstract])) OR (Taijiquan[Title/Abstract])) OR (T'ai Chi[Title/Abstract])) OR (Tai Ji Quan[Title/Abstract])) OR (Ji Quan, Tai[Title/Abstract])) OR (Quan, Tai Ji[Title/Abstract])) OR (Baduanjin[Title/Abstract])) OR (Yijinjing[Title/Abstract])) OR (Wuqinxi[Title/Abstract])) OR (Qigong[Title/Abstract])))) AND (("Accidental Falls"[Mesh]) OR (((((((((Accidental Falls[Title/Abstract]) OR (Falls, Accidental[Title/Abstract])) OR (Accidental Fall[Title/Abstract])) OR (Fall, Accidental[Title/Abstract])) OR (Falling[Title/Abstract])) OR (Falls[Title/Abstract])) OR (Slip[Title/Abstract] AND Fall[Title/Abstract])) OR (Fall[Title/Abstract] AND Slip[Title/Abstract])) OR (Balance[Title/Abstract])))
