# Supplementary material for: Effectiveness of traditional mind-body exercises on fall risk test scores and balance in older adults: A systematic review and meta-analysis
Source: Medicine (Baltimore). 2026 Oct 16;105(42):e51150. doi: 10.1097/MD.0000000000051150 (PMC13681109; doi:10.1097/MD.0000000000051150)
Supplement: Supplementary file 2 [file medi-105-e51150-s003.docx]

Table S2 GRADE evidence profile for fall risk and balance

| **Evidence item** | **Fall Risk Test scores Lower scores favor intervention** | **Berg Balance Scale scores Higher scores favor intervention** |
| --- | --- | --- |
| **Studies and design** | 7 RCT results | 24 RCT results |
| **Reported sample size** | 366 participants | 3,437 participants |
| **Pooled effect (95% CI)** | SMD −0.56 (−0.84 to −0.28) | WMD +2.60 points (+1.47 to +3.72) |
| **Heterogeneity** | I² = 56.2%; P = 0.033 | I² = 95.6%; P < 0.001 |
| **Risk of bias** | Serious (−1) | Serious (−1) |
| **Inconsistency** | Serious (−1) | Very serious (−2) |
| **Indirectness** | Not serious | Not serious |
| **Imprecision** | Not fully assessable | No additional downgrade |
| **Publication bias** | No additional downgrade Egger P = 0.092; | No additional downgrade  Egger P = 0.914; bias not excluded |
| **Overall certainty** | **Low** | **Very low** |
| **Interpretation** | Mean score was 0.56 SD lower (95% CI 0.28 to 0.84 lower). | Mean score was 2.60 points higher (95% CI 1.47 to 3.72 higher). |
